# Introduction of rural psychiatry posting in MD curriculum: A qualitative study on residents’ perspective

**DOI:** 10.1192/j.eurpsy.2023.1926

**Published:** 2023-07-19

**Authors:** K. Sadh, N. Kumar C, V. Basavaraju, S. H N, M. Narayana, S. Bada Math, P. S Chandra

**Affiliations:** 1 Department of Psychiatry, Jawaharlal Nehru Medical College, Datta Meghe Institute of Higher Education and Research, Wardha; 2 Department of Psychiatry, National Institute of Mental Health and Neuro Sciences (NIMHANS), Bengaluru, India

## Abstract

**Introduction:**

A 15-day compulsory rotatory rural psychiatry posting was introduced into the MD psychiatry curriculum at NIMHANS to orient trainees to the functioning of community mental health services.

**Objectives:**

To capture the views and subjective experiences of the 32 residents posted in rural psychiatry services under District Mental Health Program (DMHP) using qualitative interviews.

**Methods:**

In-depth qualitative interviews were conducted to understand the residents’ experience in various aspects of the community psychiatry posting. The interviews were audiotaped and later, transcribed. Thematic analysis of transcripts was done.

**Results:**

The analyzed data was converted into 41 codes and 12 themes. The themes related to positive experiences were good clinical exposure and skills to practice in low-resource settings, focus on preventive mental health care, enhanced communication, administration, leadership skills, and increased empathy. After training, the residents also reported gaining insights into the attitude of policy-makers and increased interest and confidence to practice in a rural setting. The themes highlighting the perceived challenges ranged from personal reasons, such as food or transportation, to professional ones like stigma, limited resources, a burdensome amount of paperwork, limited availability of psychotropics, and communication barriers. Residents who expressed interest in practicing rural psychiatry in the future cited a good wage, higher levels of self-satisfaction, confidence, and an emotional connection to their native place as motivating factors. Those who did not want to join DMHP had concerns such as not having adequate skills for working in a low resource setting, compromised basic needs, superiors not being sensitive to mental health issues, additional non-psychiatric work, job instability and lack of academic and research opportunities.

**Conclusions:**

The posting to nearby DMHP centers was feasible and contributed positively to the training experience of the residents. Positive experiences, challenges, and other lessons learned by these residents could help them plan their career in rural psychiatry. It was found that both the residents and the DMHP team contributed to each other’s growth. This posting was likely to boost residents’ confidence to work in rural settings and could also aid in easing the crisis of lack of community-based mental health experts. The authors advocate for the national implementation of such rural psychiatry posting.

**Disclosure of Interest:**

None Declared

